# Nanogel-Based Delivery System for Lemongrass Essential Oil: A Promising Approach to Overcome Antibiotic Resistance in *Pseudomonas aeruginosa* Infections

**DOI:** 10.3390/gels9090741

**Published:** 2023-09-13

**Authors:** Mohammed F. Aldawsari, Ahmed I. Foudah, Pinki Rawat, Aftab Alam, Mohamad Ayman Salkini

**Affiliations:** 1 Department of Pharmaceutics, College of Pharmacy, Prince Sattam Bin Abdulaziz University, Al Kharj 11942, Saudi Arabia; moh.aldawsari@psau.edu.sa; 2 Department of Pharmacognosy, College of Pharmacy, Prince Sattam Bin Abdulaziz University, Al Kharj 11942, Saudi Arabia; a.foudah@psau.edu.sa (A.I.F.); a.alam@psau.edu.sa (A.A.); 3 Prabha Harjilal College of Pharmacy and Paraclinical Sciences, Chak Bhalwal, Jammu 181122, India; drpnkrawat@gmail.com

**Keywords:** antibiotic resistance, *Pseudomonas aeruginosa*, lemongrass essential oil, nanogels

## Abstract

The emergence of antibiotic-resistant strains of *Pseudomonas aeruginosa* (*P. aeruginosa*) presents a substantial obstacle in medical environments. To effectively tackle this problem, we suggest an innovative approach: employing a delivery system based on nanogels to administer lemongrass essential oil (LGO). Developed PVA and PLGA nanoparticle formulation efficiently encapsulates LGO with 56.23% encapsulation efficiency by solvent extraction technique, preserving stability and bioactivity. Nanogel: 116 nm size, low polydispersity (0.229), −9 mV zeta potential. The nanogel’s controlled release facilitated targeted LGO delivery via pH-controlled dissolution. Pure LGO had the highest release rate, while LGO-NP and LGO-NP-CG exhibited slower rates. In 15 h, LGO-NP released 50.65%, and LGO-NP-CG released 63.58%, releasing 61.31% and 63.58% within 24 h. LGO-NP-CG demonstrated superior antioxidant activity, a lower MIC against *P. aeruginosa*, and the most potent bactericidal effect compared to other formulations. This underscores the versatile efficacy of LGO, suggesting its potential to combat antibiotic resistance and enhance treatment effectiveness. Moreover, employing a nanogel-based delivery approach for LGO offers an efficient solution to combat drug resistance in *P. aeruginosa* infections. By employing strategies such as nanogel encapsulation and controlled release, we can enhance the effectiveness of LGO against antibiotic-resistant strains. This study establishes a robust foundation for exploring innovative approaches to treating *P. aeruginosa* infections using nanomedicine and paves the way for investigating novel methods of delivering antimicrobial drugs. These efforts contribute to the ongoing battle against antibiotic resistance.

## 1. Introduction

The emergence of drug-resistant microbes, such as *Pseudomonas aeruginosa* (*P. aeruginosa*), has been driven by its adaptable nature and genetic makeup, enabling it to thrive in various environments. This bacterium poses a significant threat in both natural and human-made settings, causing infections that range from mild to severe, particularly in immunocompromised individuals. Given its prevalence and potential health risks, addressing *P. aeruginosa* infections is crucial, especially for immunocompromised individuals [1,2]. From sudden-onset to prolonged, *P. aeruginosa* may cause restricted and body-wide infections. This bacterium, especially in hospital institutions, causes many infections. Cystic fibrosis patients can have recurrent lung infections, burn injuries, and urinary tract infections. Severe cases may include bloodstream, pneumonia, and multi-organ infections [3]. *P. aeruginosa* infections pose a significant concern due to the bacteria’s resistance to antibiotics, making treatment tough. It has various ways to resist drugs, like producing enzymes, expelling antibiotics, changing its outer membrane, and forming biofilms [4,5]. It can also gain resistant genes from other bacteria. This leads to fewer treatment options, more infections, and higher deaths. To fight this, we need new drugs from research, strict infection control, and careful antibiotic use [6,7].

Certainly, the issue of antibiotic resistance caused by *P. aeruginosa* can potentially be addressed using the utilization of essential oils like lemongrass essential oil (LGO). LGO derived from plants, these oils consist of a unique composition comprising various bioactive compounds. Frequently, these chemical components demonstrate an extensive range of antibacterial effects and possess efficacy against bacteria that have developed resistance to traditional antibiotics, including *P. aeruginosa* [8,9]. The intricate makeup of essential oils consists of a variety of bioactive compounds, including but not limited to terpen. Essential oils are composed of a diverse range of bioactive chemical compounds, including terpenes, phenols, and aldehydes. This intricate composition provides a wide variety of antimicrobial agents in each oil [10,11,12]. In order to combat antibiotic-resistant bacteria like *P. aeruginosa*, essential oils possess an advantageous trait known as chemical diversity. This attribute enhances their ability to fight against these resilient microorganisms. The unique composition of bioactive constituents found in essential oils may work together synergistically, leading to a more potent antibacterial effect and the potential for overcoming bacterial resistance mechanisms. Furthermore, an additional benefit that essential oils offer is their capacity to simultaneously address multiple mechanisms of bacterial resistance. Unlike traditional antibiotics, which tend to focus on specific targets, the varied bioactive compounds in essential oils have the ability to interact with different components and pathways within bacterial cells. This versatile approach presents a challenge for bacteria in developing resistance against essential oils when compared to antibiotics that target only one aspect of microbial activity [13,14].

Although LGO has exhibited potential as an effective antimicrobial agent, its application encounters certain challenges, including low stability, rapid evaporation, and limited ability to penetrate bacterial biofilms [15,16]. Although the use of LGO nanoparticles as antibacterial agents has shown promise, there are several challenges that hinder their direct application. These include problems related to stability, evaporation, and limited penetration into bacterial biofilms. However, nanoparticle technology offers a potential solution by encapsulating the essential oil within nanoparticles. This innovative approach not only addresses some of these issues but also improves stability and controlled release and enhances the ability to penetrate bacterial biofilms [17,18]. Nevertheless, there exists a deficiency in our understanding regarding the efficient delivery of these nanoparticles to the specific site of infection [19]. Nanostructures of these oils counter volatility and instability caused by factors like temperature and UV radiation. Nanostructures can enhance the preservation, bioavailability, and biocompatibility of essential oils. Nano-encapsulation, especially in lipid-based (liposomes, solid lipid nanoparticles, etc.) and polymer-based forms, shows promise [20]. One potential solution comes in the form of nanogels, which can encapsulate and preserve nanoparticles composed of essential oils. This enables them to be specifically delivered to the site of infection and released over an extended period. By utilizing nanogels, targeted distribution, and prolonged release are made possible for essential oil nanoparticles [21,22]. By addressing this research deficiency, there is an opportunity to significantly enhance the therapeutic properties of LGO nanoparticles and expedite the progress toward more potent treatments for *P. aeruginosa* infections.

**Scheme 1:**
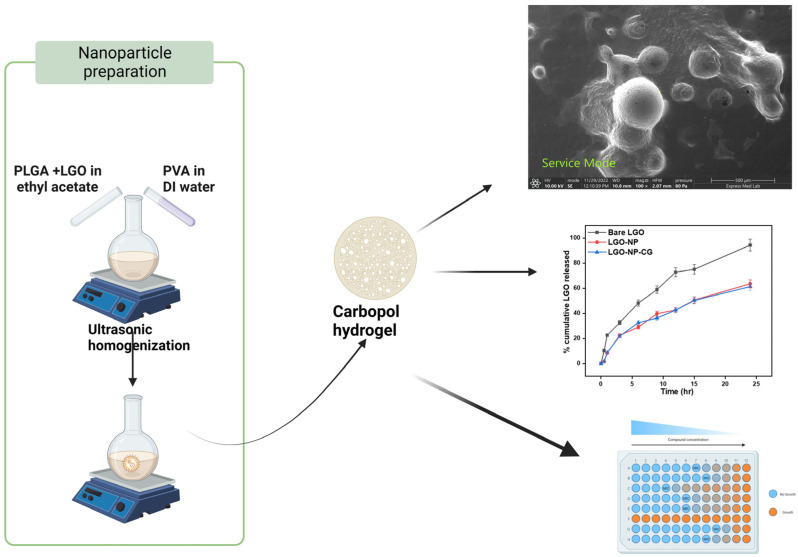
Workflow of the experimental work.

The aim of this study is to prepare the formulation of LGO nanoparticles through solvent emulsification-diffusion technique. The study aims to optimize nanogel formulations, assess their release kinetics, and determine their efficacy in delivering LGO nanoparticles to address antibiotic resistance in *P. aeruginosa*. In this context, solvent residues from the emulsification process can impact the quality and properties of the nanogels. Residual solvents might affect the stability, biocompatibility, and drug-loading capacity of the nanogels. Therefore, meticulous attention must be paid to the removal of solvent residues during the formulation process to ensure the safety and effectiveness of the nanogels for targeted and controlled release of LGO nanoparticles at infection sites. The successful achievement of this research work will contribute to improving the therapeutic potential associated with LGO nanoparticles, thus promoting more effective treatments for *P. aeruginosa* infections. The complete workflow is shown in Scheme 1.

## 2. Results and Discussion

LGO-NPs were prepared utilizing a solvent emulsification-diffusion technique, incorporating two biocompatible polymers, namely PVA and PLGA. The choice of employing PVA and PLGA as biocompatible materials facilitated the consistent and controlled release of LGO from the nanoparticles. Using the solvent emulsification-diffusion approach, an oil-in-water emulsion was formed, leading to the development of stable nanoparticles with uniform properties. Furthermore, using the lyophilization process, LGO-NPs achieved long-term stability, enabling their suitability for storage and characterization purposes.

### 2.1. Particle Size, Size Distribution, Zeta Potential, and Morphology

**Figure 1:**
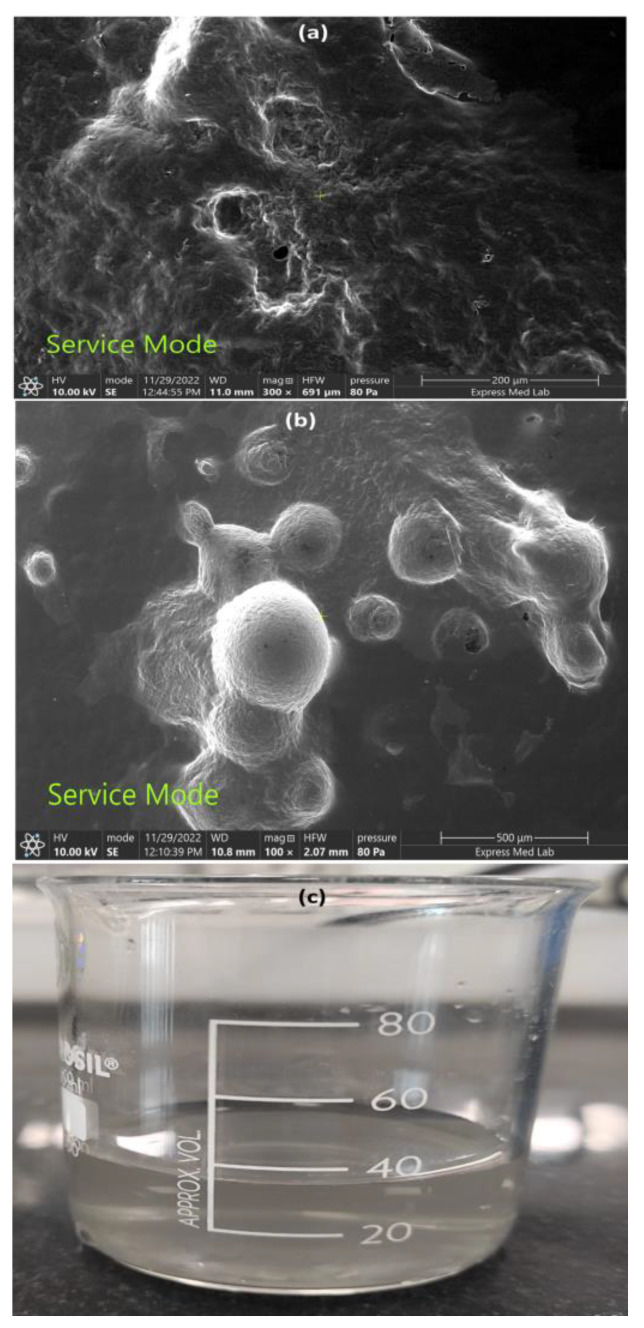
(**a**,**b**) FESEM image of LGO-NPs; (**c**) Final LGO-NP formulation.

The characterization data unveils that LGO-NPs possess a particle size of 116 nm, along with a low polydispersity index (PDI) value of 0.229 and a negative zeta potential of −9 mV. The modest particle size implies improved permeability across biological barriers and enhanced interaction with target cells. Concurrently, the low PDI signifies a narrow size distribution, indicating uniformity in particle size [23]. The negative zeta potential observed for LGO-NPs points to the presence of negatively charged functional groups on their surface. This charge is pivotal in electrostatically stabilizing the nanoparticles, averting aggregation when dispersed in solution [24]. Further insight into the physical attributes and surface morphology of LGO-NPs was obtained using SEM imaging (Figure 1a,b) at two different magnifications: 300× and 100×. The nanoparticles exhibit a spherical shape accompanied by a smooth surface. Notably, certain nanoparticles display an adhesive tendency, resulting in agglomeration. This stickiness can be attributed to a polymer coating enveloping their surfaces. This polymer coating serves a dual purpose: shielding the nanoparticles from degradation and modulating the release of encapsulated drugs. However, this adhesive characteristic potentially influences other nanoparticle attributes, encompassing size, configuration, and solubility [25]. This aggregation may impede their efficient delivery to the designated target site. Furthermore, the stickiness of the nanoparticles influences the kinetics of drug release [26]. The adhesive nanoparticles might exhibit a comparatively slower drug release profile when contrasted with their non-adhesive counterparts. The image of the final formulation is shown in Figure 1c.

### 2.2. %. Encapsulation Efficiency

The encapsulation efficiency of nanoparticles plays a crucial role in the formulation process as it represents how effectively the nanoparticles can capture and retain the desired substance. Generally, higher values of encapsulation efficiency indicate superior performance in terms of trapping and stabilizing the loaded substance within the nanoparticles. The efficiency of encapsulating LGO in PVA and PLGA nanoparticles was found to be 56.23 ± 1.99% (*n* = 3) using solvent extraction technique. This value represents the percentage of LGO that was successfully enclosed within the PVA and PLGA nanoparticles, compared to the total amount used during the process of nanoparticle formation. The remaining percentage, which amounts to 43.77%, denotes the fraction of LGO that was not successfully encapsulated or might have been lost during the preparation process. Our findings are consistent with the prior study conducted by Ramirez et al., in which they also utilized these two polymers to formulate nanoparticles [27]. Several factors could contribute to this outcome, including incomplete encapsulation of the oil within nanoparticles, potential leakage from the nanoparticles themselves, or losses occurring throughout purification steps. In the context of our encapsulation technique for essential oils using polymer-based nanoparticles, the potential presence of residual solvents demands careful consideration. Highlighting the significance of evaluating residual solvents in encapsulation technology, a study conducted by Vora et al. demonstrated this concern. In their study, they undertook a series of wash steps to eliminate surface-attached PVA from microspheres before freeze-drying. The analysis revealed that the residual PVA content in the freeze-dried cholecalciferol-loaded microspheres (CL-MS) was measured at 14 μg per 100 mg of formulation. This negligible residue reinforces the safety and viability of our encapsulation method for potential parenteral applications [28].

### 2.3. Characterization of LGO-NP-CG

Physical Properties: The hydrogel containing LGO-NP-CG exhibited a gel-like consistency characterized by its smooth and uniform texture. It is worth noting that the addition of LGO-NPs did not result in any significant changes to the overall appearance or coloration of the hydrogel, suggesting excellent dispersion and compatibility between the nanoparticles and gel matrix.

pH: The acidity or alkalinity of the Carbopol hydrogel containing LGO-NP was assessed by analyzing its pH level. The pH value plays a crucial role in determining the stability, compatibility, and overall effectiveness of formulations based on hydrogels. The pH value of the Carbopol hydrogel containing LGO-NPs was determined to be 6.0 using measurement analysis. This specific pH value holds significance as it provides valuable insights into the characteristics of the hydrogel and can impact the release kinetics of LGO-loaded nanoparticles within its matrix. The maintenance of an appropriate pH level is crucial in order to guarantee the stability and compatibility with living organisms of hydrogels used for various purposes, including topical applications or the delivery of drugs via the skin. For instance, by determining that the LGO-NP incorporated Carbopol hydrogel exhibits a pH value of 6.0, This initial finding serves as a foundation upon which further refinements can be made to optimize and customize the composition according to specific needs, thereby maximizing its therapeutic efficacy.

Viscosity: The viscosity of Carbopol (0.5%) hydrogel containing LGO-NP is a critical determinant affecting its overall efficacy and usage. The hydrogel displays a significant level of thickness and resistance to flow, with an average viscosity range of approximately 40,000 cP. This slow-flowing behavior plays a pivotal role in specific applications that require sustained release or prolonged interaction with the intended target area.

Spreadability capacity: The spreadability of the LGO-NP-CG formulation was found to be 213.2 ± 0.7 mm^2^, which is a measure of its ability to spread and cover a surface when applied. A higher value for spreadability indicates that the hydrogel can cover a larger area with less product. This property allows for smooth and even application over the entire surface. The observed spreadability is influenced by several factors, including the formulation’s rheological properties and consistency.

### 2.4. In Vitro Release Study

**Figure 2:**
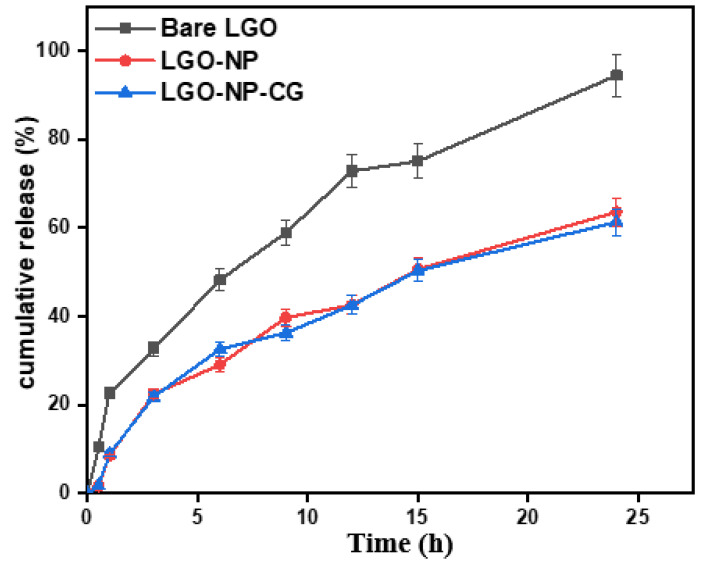
Comparative in vitro LGO release study of bare LGO, LGO-NP, and LGO-NP-CG.

The provided data represents an in vitro LGO release study conducted at pH 6, comparing the LGO release profiles of bare LGO, LGO-NP, and LGO-NP-CG over a specified time period in hours, as shown in Figure 2. At the beginning of the study (0 h), all three formulations exhibited no drug release, as indicated by the values of 0 for each formulation. However, as the study progressed, the drug release profiles started to differ among the formulations. After 0.5 h, the Bare LGO formulation showed a drug release of 10.39%, while LGO-NP and LGO-NP-CG released lower amounts of the drug, with values of 1.71% for both formulations. At the 1 h mark, the drug release increased for all three formulations. Bare LGO had the highest release at 22.69%, while LGO-NP and LGO-NP-CG showed drug releases of 8.48% and 8.93%, respectively. As the study progressed to 3 h, the drug release continued to increase for all formulations. Bare LGO reached a release of 32.66%, while LGO-NP and LGO-NP-CG exhibited drug releases of 22.49% and 21.81%, respectively. At 6 h, the drug release values further increased. Bare LGO showed the highest release at 48.22%, while LGO-NP and LGO-NP-CG had drug releases of 29.08% and 32.60%, respectively. After 9 h, the drug release continued to rise. Bare LGO exhibited a release of 58.92%, while LGO-NP and LGO-NP-CG had drug releases of 39.76% and 36.29%, respectively. At the 12 h mark, Bare LGO had released 72.84% of the drug, while LGO-NP and LGO-NP-CG showed drug releases of 42.59% and 42.56%, respectively. The drug release profiles at 15 and 24 h showed a similar trend. Bare LGO had the highest drug release percentages, reaching 75.11% and 94.43% at 15 and 24 h, respectively. LGO-NP and LGO-NP-CG exhibited lower drug releases, with values of 50.65% and 63.58% at 15 h and 61.31% and 63.58% at 24 h, respectively. The comprehensive analysis of the data underscores distinct patterns in drug release among the three formulations: Bare LGO, LGO-NP, and LGO-NP-CG. Notably, the release profile of Bare LGO exhibited a rapid and substantial release in comparison to the LGO-NP and LGO-NP-CG formulations. On the other hand, the LGO-NP and LGO-NP-CG formulations demonstrated sustained drug release behaviors, suggestive of the presence of nanoparticles and their influential role in the release kinetics. These findings serve to emphasize the pivotal role that formulation and coating strategies play in dictating the rate and extent of drug release. The observed sustained release patterns within the LGO-NP and LGO-NP-CG formulations can be attributed to the encapsulation mechanisms employed, allowing for a controlled diffusion of the drug over time. This controlled and sustained drug release carries significant implications for various applications. In particular, the ability to modulate the release of LGO using formulation strategies holds promise for diverse therapeutic applications. The sustained release kinetics exhibited by LGO in nanoparticle-based formulations could potentially translate to extended therapeutic effects. This is especially relevant in scenarios where targeted and prolonged drug delivery is crucial for therapeutic success. Our study aligns with the findings of a prior investigation by Elsewedy et al. In their work, they successfully formulated brucine-loaded nanoparticles using PLGA. Notably, the in vitro drug release rate remained below 99.1% even after 168 h, indicating sustained and controlled release characteristics [29].

In this study, the evaluation of drug release kinetics was conducted using the application of five distinct kinetic models, namely Zero Order, Higuchi, First Order, Korsmeyer-Peppas, and Hixson-Crowell, as outlined in Table 1. The purpose of this analysis was to comprehensively understand the release profiles of the formulations by employing various mathematical models, each of which is grounded in specific equations and underlying assumptions. Table 1 serves as a comprehensive resource that presents a compilation of processing methods, each accompanied by the corresponding outcomes achieved. This compilation aims to elucidate the diverse impacts of distinct processing approaches on the intended outcomes. Each kinetic model, including the Higuchi model, uses its own distinct parameters and assumptions to analyze the drug release data, contributing to a holistic understanding of the release mechanisms at play. The table further provides insight into the quality of fit between the experimental data and the predictions generated using each kinetic model. Specifically, it presents the correlation coefficients (R^2^) for each formulation—bare LGO (R^2^ = 0.9938), LGO-NPs (R^2^ = 0.9878), and LGO-NP-CG (R^2^ = 0.9869). Intriguingly, across all three formulations, a close alignment with the Higuchi model for drug release was observed, substantiated using the high R^2^ values. In a prior study, Pourtalebi Jahromi et al. conducted a comparison of various models to assess the kinetics of drug release from nanoparticles based on PLGA. The majority of the datasets exhibited the Weibull model with the highest R^2^ values [30].

### 2.5. Antioxidant Activity

The IC_50_ values, which represent the concentration of the formulation required to scavenge 50% of the DPPH free radicals, were determined for each formulation as given in Table 2. The results showed that all three formulations exhibited significant antioxidant activity, as indicated by their low IC_50_ values. Among the formulations, LGO-NP-CG demonstrated the highest antioxidant activity, with an IC_50_ value of 0.153 mg/mL. This suggests that LGO-NP-CG was the most effective in scavenging DPPH free radicals, requiring the lowest concentration to achieve a 50% reduction in DPPH activity. LGO-NPs also exhibited notable antioxidant activity, with an IC_50_ value of 0.271 mg/mL. Although slightly higher than LGO-NP-CG, it still displayed strong antioxidant potential. Bare LGO, without the nanoparticle or coating, showed the least potent antioxidant activity among the three formulations, with an IC_50_ value of 0.557 mg/mL. At the same time, the IC_50_ value of standard, i.e., of gallic acid, is 0.088 mg/mL. The nanoparticles and the coating might have contributed to increased stability, improved bioavailability, and prolonged release of antioxidant compounds from the formulations, leading to enhanced scavenging of DPPH free radicals.

### 2.6. Minimum Inhibitory Concentration in P. aeruginosa

The results indicate that both LGO-NPs and LGO-NP-CG exhibited lower MIC values compared to Bare LGO, suggesting a higher antimicrobial activity for the nanoparticle-based formulations, as given in Table 3. Amikacin, a known antibiotic, exhibits a significantly lower MIC value of 0.0042 mg/mL. LGO-NPs displayed a MIC value of 3.4 mg/mL, while LGO-NP-CG demonstrated an even lower MIC value of 3.2 mg/mL. These results indicate that a lower concentration of the nanoparticle formulations was required to inhibit the growth of *P. aeruginosa*. On the other hand, Bare LGO had a higher MIC value of 10 mg/mL, indicating that a higher concentration of the unencapsulated LGO was needed to achieve the same inhibitory effect on *P. aeruginosa*. This underscores the strong antimicrobial potency of amikacin in comparison to the LGO-based formulations. These findings suggest that the nanoparticle formulations, particularly LGO-NPs and LGO-NP-CG, have the potential to serve as effective antimicrobial agents against *P. aeruginosa*. The lower MIC values for these formulations indicate their stronger inhibitory effects on the growth of the bacterium, highlighting their potential applications in combating *P. aeruginosa* infections.

### 2.7. Time-Kill Assay

**Figure 3:**
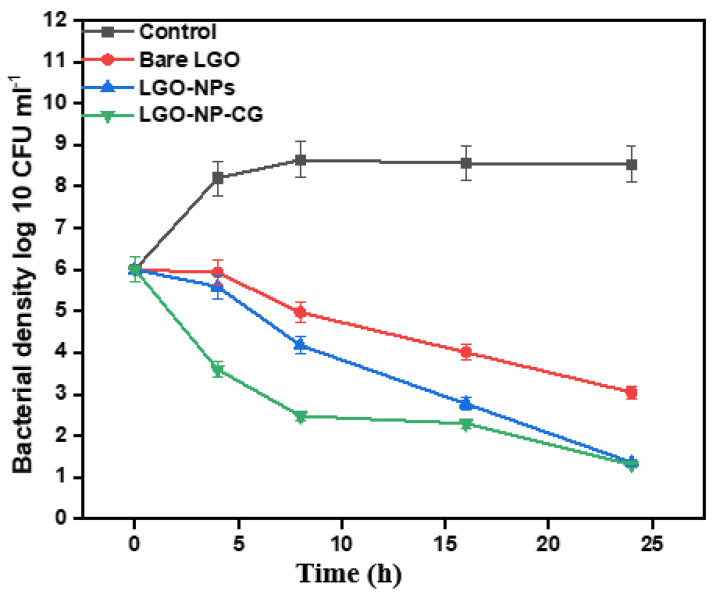
Time-kill assay of LGO, LGO−NP, and LGO−NP−CG against *P. aeruginosa*.

In order to assess the effectiveness of various formulations, including Bare LGO, LGO-NPs, and LGO-NP-CG, in eliminating *P. aeruginosa*, a kill kinetic assay was performed, as shown in Figure 3. The assay aimed to determine the time required for each formulation to display a bactericidal effect at concentrations equivalent to twice the minimum inhibitory concentration (2× MIC) for each formulation. Notably, results from this experiment indicated that formulations containing LGO exhibited significant reductions in *P. aeruginosa* viability compared to the control group. The bacterial cell count of bare LGO gradually decreased over time, with log CFU/mL values declining from 5.93 at 4 h to 3.05 at 24 h. However, LGO-NPs exhibited enhanced bactericidal activity compared to Bare LGO, resulting in a more rapid reduction in the cell count. The log CFU/mL values for LGO-NPs decreased from 5.59 at 4 h to an astonishingly low value of 1.36 after 24 h. Significantly, of all the tested formulations, LGO-NP-CG exhibited the most powerful bactericidal effect and resulted in the highest decrease in bacterial cell count observed throughout the entire experiment. This suggests that all three formulas displayed bactericidal activity against *P. aeruginosa* at concentrations 2 times higher than their minimum inhibitory concentration. Notably, LGO-NP-CG demonstrated superior potency compared to both LGO-NPs and Bare LGO in terms of reducing bacterial cell count over time. These findings highlight that among these formulations, LGO-NP-CG showed outstanding efficacy as a potential treatment strategy for combating *P. aeruginosa* infections. The superior bactericidal effects observed in the nanoparticle formulations can be credited to enhanced delivery, prolonged contact time, and potential synergistic effects between the nanoparticles and the antimicrobial compounds found in LGO. To conclude, the kill kinetic assay conducted verified the efficacy of the formulations in decreasing *P. aeruginosa* viability over a period of time. Additional investigation is required to unveil and understand the mechanisms responsible for the amplified bactericidal activity witnessed in these nanoparticle formulations.

## 3. Conclusions

We successfully encapsulated LGO while maintaining its stability and bioactivity by formulating nanoparticles using PVA and PLGA. The resulting nanogel exhibited favorable physicochemical properties, facilitating precise LGO delivery to the target site. The controlled release characteristics of this nanogel system amplified LGO’s potency against antibiotic-resistant strains. Comparative investigations demonstrated that LGO-loaded nanoparticles formulated with carbopol showcased elevated antioxidant activity and a lower minimum inhibitory concentration against *P. aeruginosa* compared to other formulations. Notably, LGO-NP-CG displayed remarkable bactericidal potential, leading to a significant reduction in bacterial cell count. These findings underscore LGO’s versatile attributes, suggesting its potential in countering antibiotic resistance and optimizing treatment outcomes. Moreover, leveraging nanogel-based delivery strategies for administering LGO holds promise in combating drug resistance in *P. aeruginosa* infections. This study lays a robust foundation for innovative nanomedicine approaches in treating *P. aeruginosa* infections, fostering research into novel antimicrobial delivery methods that contribute to the global fight against antibiotic resistance. Via harnessing nanogel encapsulation and controlled release capabilities, we can enhance LGO’s effectiveness, facilitating more efficient treatments for antibiotic-resistant *P. aeruginosa* strains.

## 4. Materials and Methods

Lemongrass essential oil was obtained from Shiva Exports India. Poly (vinyl alcohol) (PVA) (CAS-9002-89-5; average Mw ~100,000 by GPC) and poly (D,L-lactide-co-glycolide) (PLGA) (CAS-34346-01-5; average Mw 6000–10,000) were purchased from Sigma–Aldrich. Carbopol 940 LR (CAS-940 9003-01-4; average Mw 72.06 g/mol) procured from Bangalore Fine Chemicals, Bengaluru, Karnataka, India. *P. aeruginosa* (ATCC-11229) was obtained from the Department of Pharmaceutics, College of Pharmacy, Prince Sattam bin Abdulaziz University, Al-Kharj, Saudi Arabia. 96-well plate for culturing the cells was directly procured from Corning, NY, USA. There was no additional purification of the solvents and compounds of analytical grade. All solutions were prepared using deionized (DI) water. All other chemicals used in this study were of analytical grade and were used without the need for additional purification.

### 4.1. Preparation of Nanoparticles

In this study, LGO-loaded nanoparticles were developed using the solvent emulsification-diffusion method. This technique incorporated biocompatible polymers, PVA, and PLGA. The procedure began by combining carefully prepared saturated aqueous and organic phases. Specifically, a solution containing 350 mg PLGA in 10 mL ethyl acetate, and after that, LGO was added to it. It was mixed properly. Concurrently, a 1% (*w*/*v*) PVA solution was dissolved in 40 mL of distilled water, forming the aqueous phase. Controlled introduction of the organic phase into the aqueous phase was carried out under ice-cold conditions. This was achieved using an ultrasonic homogenizer operating at 100 watts for five minutes to ensure constant and vigorous emulsification, resulting in an oil-in-water emulsion. The emulsion was diluted with distilled water and magnetically stirred to facilitate solvent diffusion. After freezing and lyophilization, the nanosuspension was stored in a sealed container at 4 °C [31,32]. The entire formulation process was conducted in triplicate to ensure consistency and reproducibility of the results.

### 4.2. Particle Size and Size Distribution

Dynamic light scattering (DLS) is a technique that uses the Brownian motion of particles to measure their size [33]. Collisions with solvent molecules caused particle Brownian motion. The timeframe of Brownian motion was influenced by the particle size. To this, nanoparticles were dispersed in DI water. It was crucial to ensure that the sample was well-dispersed and free of aggregates and contaminants. Subsequently, the sample was analyzed in a cuvette using a DLS apparatus Zetasizer Nano ZS, model ZEN3500, to conduct size analysis on nanoemulsions (Malvern Instruments, Malvern, UK) was used. The DLS analysis involved observing variations in scattered light intensity to identify particle size and distribution.

### 4.3. Surface Morphology and Shape

Field Emission Scanning electron microscopy (FESEM) image of LGO-NPs was taken in a Quattro S EDS: Thermo Scientific -Ultra Dry Manufacturing, Grand Island, NY, USA operated under 5 kV accelerating voltage. The dispersion solution of LGO-NPs was drop cast on a carbon tape and dried at 60 °C for 6 h. Further, the sample deposited carbon tape was mounted on the sample holder of FESEM without any coating [34].

### 4.4. %. Encapsulation Efficiency

The quantification of the encapsulation efficiency (EE %) for essential oils was conducted using solvent extraction technique by modifying previously established methods [35]. To determine the EE %, LGO-NPs were specifically introduced into 5 mL of hydrochloric acid solution with a concentration of 1 M and subsequently incubated in a water bath for 30 min. For comparative purposes, a control or blank sample was prepared by replacing the dispersion containing EOs with an appropriate volume of water. The entrapment effectiveness and loading capacity of LGOs were calculated employing the following formula at a wavelength setting corresponding to 396 nm:

(1) $$\%\;EE=\frac{\left(Total\;amount\;of\;LGOs\;added\;-\;Amount\;of\;free\;LGOs\;in\;supernatent\right)}{Total\;amount\;of\;LGOs\;added}\times 100$$

### 4.5. Preparation of LGO-NP Incorporated Carbopol Hydrogel

The hydrogel that contained LGO-NPs and Carbopol was prepared by following a step-by-step process in which distilled water was used. To start, a specific amount of Carbopol (0.5% *w*/*w*) was dispersed into the distilled water while being continuously stirred magnetically until complete hydration and gel formation took place. Subsequently, the dispersion was left undisturbed for a designated period to ensure proper gelation occurred. Afterward, the desired concentration of LGO-NPs was introduced into the Carbopol gel via careful homogenization using a magnetic stirrer to achieve an even distribution within the hydrogel matrix. In order to ensure uniform dispersion of the nanoparticles within the hydrogel, a careful and precise method was employed. This approach aimed at improving the therapeutic efficacy of the loaded nanoparticles throughout the gel matrix. Subsequently, an evaluation was conducted to assess various physical properties of interest, including color, appearance, pH levels, and viscosity. These analyses played a significant role in providing comprehensive insights into the overall characteristics and appropriateness of this particular formulation for its intended application. By adhering to strict control over each step involved in formulating this hydrogel and meticulously assessing its resulting properties using rigorous evaluations, this study aspired not only towards optimizing such formulations but also enhancing their potential utility as effective systems for drug delivery or other relevant therapeutic interventions [36,37]. The LGO-NP incorporated Carbopol hydrogel (LGO-NP-CG) was subjected to comprehensive evaluation to assess its suitability for potential therapeutic applications.

Physical Properties: The color and appearance of the hydrogel were visually checked. Any observable changes in color or presence of particles were noted, indicating the stability and uniform dispersion of LGO-NPs within the hydrogel matrix [38].

pH: The pH of the hydrogel was measured using a pH meter to determine its acidity or alkalinity. This parameter is crucial as it can affect the compatibility and tolerability of the hydrogel on the target site of the application. The pH range was compared with the physiological pH of the intended application site to ensure optimal conditions.

Viscosity: Moreover, the hydrogel’s viscosity was assessed using an appropriate viscometer. The degree of viscosity plays a crucial role in determining how the hydrogel flows and spreads, affecting its application ease and ability to penetrate into the intended area effectively. By measuring its viscosity, valuable information regarding the hydrogel’s consistency and rheological properties can be obtained.

Spreadability capacity: The ability of the experimental hydrogels to spread evenly across the skin surface was assessed by evaluating their spreadability capacity. To perform this evaluation, a spreadability apparatus consisting of two parallel glass plates was utilized. A specific quantity of the hydrogel being tested was deposited onto the lower glass plate and then covered with the upper plate. Subsequently, an established weight was applied to the upper plate for a set duration. Following this time period, the upper plate was lifted, and measurements were taken to determine the diameter of spreading exhibited by the hydrogel. This test offered a way to determine the ability of the hydrogel to spread. The larger the diameter of the spread, the better its spreadability, indicating that it could effectively cover a wider area. To ensure accuracy and reproducibility, three experiments were conducted for each formulation of hydrogel. The findings from evaluating the spreadability played an important role in assessing its physical properties and determining its suitability for topical use.

### 4.6. In Vitro Drug Release Study

The investigation of the release behavior of the nanohydrogel formulated for treating *P. aeruginosa* infection was performed using the dialysis method. This study played a vital role in providing necessary insights into the drug release profile of the nanohydrogel over time. To conduct this study, a dissolution testing methodology was employed using a pH buffer as the medium for dissolution. The nanohydrogel consisted of LGO, LGO-NP, and LGO-NP-CG, and it was placed in a dialysis membrane to enable controlled drug release while creating a barrier between the hydrogel and the dissolution medium. Additionally, to mimic conditions similar to those found at *P. aeruginosa* infection sites, which typically have a pH of around 6, immersion took place in a pH-buffered solution. Selecting a pH of 6 for the in vitro drug release study in transdermal drug delivery aligns with the skin’s natural pH, enhances the relevance of the study’s findings, and provides valuable insights into the system’s behavior during real-world application. During the entire experiment, a constant stirring of the dissolution medium was maintained in order to facilitate effective mixing. At specific time intervals, samples were extracted from the dissolution medium for analysis purposes. The drug content released from the nanohydrogel formulation was analyzed using UV spectrophotometry (Shimadzu, Japan). The rationale behind opting for UV spectrophotometry rests on its compatibility with compounds exhibiting distinct UV-absorbing properties, which are often characteristics of drugs and bioactive molecules. Moreover, the widespread availability of UV spectrophotometry in laboratory settings further validates its appropriateness for evaluating the drug release dynamics and the overall efficacy of the nanohydrogel formulation within the scope of this study. Additionally, we conducted kinetic studies based on the data obtained from drug release experiments, which offer valuable insights into how drugs are released from nanohydrogel formulations. This investigation provides crucial information and understanding that aids in optimizing and comprehending different aspects of drug delivery systems.

### 4.7. Antioxidant Activity

To determine the antioxidant activity of LGO, LGO-NP, and LGO-NP-CG, the widely used 2,2-diphenyl-1-picrylhydrazyl assay can be employed. The procedure entails several steps. Initially, a stock solution of LGO is prepared in an appropriate solvent such as ethanol:water (1:1). Subsequently, this stock solution is diluted to create a range of concentrations from 0.25 mg/mL to 1.25 mg/mL for each sample using proper dilution techniques. In separate test tubes for each concentration level, one milliliter of DPPH solution and 1 mL of the respective sample solutions are combined. These mixing procedures enable further analysis of their potential antioxidant capabilities. The test tubes are then vigorously shaken and allowed to stand in the dark for a specified duration, typically around 30 min, to ensure a complete reaction. After the incubation period, the absorbance of each solution is measured using a spectrophotometer (Systronic, India) at a specific wavelength, commonly around 517 nm. To auto-zero the spectrophotometer, ethanol was used as the negative blank. The percentage of radical scavenging activity of the sample solution was determined using the following equation. A higher scavenging activity indicates the greater antioxidant potential of the sample. To maintain the accuracy and reproducibility of results, this procedure was repeated three times (in triplicate) [39].

Percentage of radical scavenging activity (% RSA) = (A sample − Ablank)/A sample × 100.

### 4.8. Minimum Inhibitory Concentration

The determination of the minimum inhibitory concentration (MIC) was conducted using sterile microtiter plates. Initially, stock solutions of bare nanogel, LGO-NP, and LGO-NP-CG were prepared in water at a concentration of 1 mg/mL to ensure complete solubilization. In the microtiter plate, 100 µL of nutrient broth and Sabouraud dextrose broth were added to wells 1 to 10, followed by the addition of 100 µL of bare nanogel, LGO-NP, and LGO-NP-CG samples to the first well. Serial dilutions were performed from well 1 to well 10, with 100 µL from well 10 being discarded. Subsequently, 100 µL of the bacterial suspension was added to all the dilution wells from well 1 to well 10. In well 11, 100 µL of the overnight bacterial suspension of *P. aeruginosa* was added, and 100 µL of sterile broth served as the positive control or growth control. In well 12, 200 µL of sterile nutrient broth and Sabouraud dextrose broth were used as the negative or sterility control. The microtiter plate was then incubated at 37 °C for 24 h. Following the incubation period, the absorbance of each well was determined using an ELISA reader with a wavelength of 640 nm. This measurement allowed for the evaluation of bacterial growth inhibition. The same procedure was repeated for each microbial strain. To ensure accuracy and reliability, each test was performed in triplicate. The concentrations of the samples and standards that inhibited 50% of bacterial growth were determined for all the tested microorganisms. By conducting the tests in triplicate and averaging the results, potential errors were minimized. This comprehensive approach provided valuable data on the MIC of bare nanogel, LGO, LGO-NP, and LGO-NP-CG against different microbial strains, including *P. aeruginosa*.

### 4.9. Time-Kill Assay

Time-kill kinetics of the chosen formulations LGO, LGO-NP, and LGO-NP-CG were determined by adapting a method from a published paper [40]. The kill kinetic assay against *P. aeruginosa* was conducted using a 2× MIC. A bacterial inoculum with a size of 1 × 10^6^ colony-forming units per milliliter (CFU/mL) was added to the assay and subsequently incubated at a temperature of 37 °C. Samples were taken from the medium at time intervals of 0, 4, 8, 16, and 24 h. The number of colony-forming units for bacterial cells was determined using analysis. To serve as a control group comparison, an additional assessment considering organisms along with dimethyl sulfoxide, without any treatment applied, was also included in the experimental setup. All assays were performed three times independently (“triplicate”). Time-kill plots expressing log10 CFU/mL values from calculated mean colony counts were generated based on these measurements.

## Figures and Tables

**Table 1:** Kinetic model was applied to analyze the dissolution study results of bare LGO, LGO-NP, and LGO-NP-CG.

Formulation	Zero Order	Higuchi	First Order	Kors-Peppas	Hixson–Crowell
Bare LGO	0.9017	0.9938	0.9746	0.6052	0.9876
LGO-NPs	0.9155	0.9878	0.9764	0.8316	0.9601
LGO-NP-CG	0.9023	0.9869	0.9663	0.8232	0.9483

**Table 2:** IC_50_ for bare LGO, LGO-NP, LGO-NP-CG, and gallic acid for DPPH free radicals (*n* = 3).

Formulation	IC_50_ (mg/mL)
Bare LGO	0.557 ± 0.003
LGO-NPs	0.271 ± 0.001
LGO-NP-CG	0.153 ± 0.004
Gallic acid	0.088 ± 0.007

**Table 3:** Comparative MIC determination of the bare LGO, LGO-NP, LGO-NP-CG, and amikacin against *Pseudomonas aeruginosa*.

Sample	MIC (mg/mL)
Bare LGO	10
LGO-NPs	3.4
LGO-NP-CG	3.2
Amikacin	0.0042

## Data Availability

The data presented in this study are available on request from the corresponding author.
